# Draft genome sequence of the male-killing *Wolbachia* strain *w*Bol1 reveals recent horizontal gene transfers from diverse sources

**DOI:** 10.1186/1471-2164-14-20

**Published:** 2013-01-16

**Authors:** Anne Duplouy, Iñaki Iturbe-Ormaetxe, Scott A Beatson, Jan M Szubert, Jeremy C Brownlie, Conor J McMeniman, Elizabeth A McGraw, Gregory D D Hurst, Sylvain Charlat, Scott L O’Neill, Megan Woolfit

**Affiliations:** 1 School of Biological Sciences, University of Queensland, 4072, Brisbane, QLD, Australia; 2 Metapopulation Research Group, The University of Helsinki, PO Box 65, Viikinkaari 1, 00014, Helsinki, Finland; 3 School of Biological Sciences, Monash University, Clayton, VIC, 3800, Australia; 4 School of Chemistry and Molecular Biosciences, University of Queensland, Brisbane, QLD, 4072, Australia; 5 School of Biomolecular and Physical Sciences, Griffith University, Brisbane, 4111, Australia; 6 Laboratory of Neurogenetics and Behavior, The Rockefeller University, 1230 York Avenue, Campus Box 63, New York, NY, 10065, USA; 7 Institute of Integrative Biology, University of Liverpool, Biosciences Building, Crown Street, Liverpool, L69 7ZB, United Kingdom; 8 UMR CNRS 5558, Laboratoire de Biométrie et Biologie Évolutive, UCB Lyon 1, Bâtiment Grégor Mendel, 43 bd du 11 novembre 1918, 69622, Villeurbanne cedex, France; 9 Institute for Molecular Bioscience, University of Queensland, 4072, Brisbane, QLD, Australia

## Abstract

**Background:**

The endosymbiont *Wolbachia pipientis* causes diverse and sometimes dramatic phenotypes in its invertebrate hosts. Four *Wolbachia* strains sequenced to date indicate that the constitution of the genome is dynamic, but these strains are quite divergent and do not allow resolution of genome diversification over shorter time periods. We have sequenced the genome of the strain *w*Bol1-b, found in the butterfly *Hypolimnas bolina*, which kills the male offspring of infected hosts during embyronic development and is closely related to the non-male-killing strain *w*Pip from *Culex pipiens*.

**Results:**

The genomes of *w*Bol1-b and *w*Pip are similar in genomic organisation, sequence and gene content, but show substantial differences at some rapidly evolving regions of the genome, primarily associated with prophage and repetitive elements. We identified 44 genes in *w*Bol1-b that do not have homologs in any previously sequenced strains, indicating that *Wolbachia*’s non-core genome diversifies rapidly. These *w*Bol1-b specific genes include a number that have been recently horizontally transferred from phylogenetically distant bacterial taxa. We further report a second possible case of horizontal gene transfer from a eukaryote into *Wolbachia*.

**Conclusions:**

Our analyses support the developing view that many endosymbiotic genomes are highly dynamic, and are exposed and receptive to exogenous genetic material from a wide range of sources. These data also suggest either that this bacterial species is particularly permissive for eukaryote-to-prokaryote gene transfers, or that these transfers may be more common than previously believed. The *w*Bol1-b-specific genes we have identified provide candidates for further investigations of the genomic bases of phenotypic differences between closely-related *Wolbachia* strains.

## Background

*Wolbachia pipientis*, a bacterial endosymbiont of a vast range of insect and other arthropod species [1], is maternally transmitted, and commonly enhances its transmission to the next host generation by modifying its hosts’ reproductive systems. Different strains of *Wolbachia* induce different modifications, including parthenogenesis, feminization of genetic males, cytoplasmic incompatibility, and male-killing [2]. Other strains ensure their transmission by becoming obligate mutualists [3], while yet others use a combination of strategies and act as moderate reproductive parasites while providing their host with benefits such as increased fecundity [4], metabolic provisioning during nutritional stress [5] or protection from pathogens [6-9].

This diversity of host effects is mirrored by the genetic diversity found between strains of *Wolbachia pipientis*. To date, the complete genomes of four *Wolbachia* strains have been sequenced and described: *w*Mel [10], *w*Ri [11], *w*Pip [12], and *w*Bm [13]. These four strains represent a range of phenotypes: wBm is an obligate mutualist, while *w*Mel, *w*Ri and *w*Pip induce cytoplasmic incompatibility and offer varying degrees of pathogen protection to their hosts. They also represent a moderate proportion of the phylogenetic diversity present in *Wolbachia*. This species has been divided into fourteen ‘supergroups’, or divergent clades, named A to M [14,15], and the complete genomes are drawn from three of these: *w*Mel and *w*Ri are A-group, *w*Pip B-group, and *w*Bm D-group. Comparison of the four strains suggests that *Wolbachia*, in contrast to obligate symbionts such as *Buchnera*, have highly flexible gene content, despite their generally small genome sizes e.g. [16]. However, the divergence between previously sequenced strains has made it difficult to characterise the tempo and mode of divergence of *Wolbachia* strains. In addition, the sheer number of differences between genomes makes it impossible to link genomic differences to any particular aspect of symbiosis.

**Figure 1:**
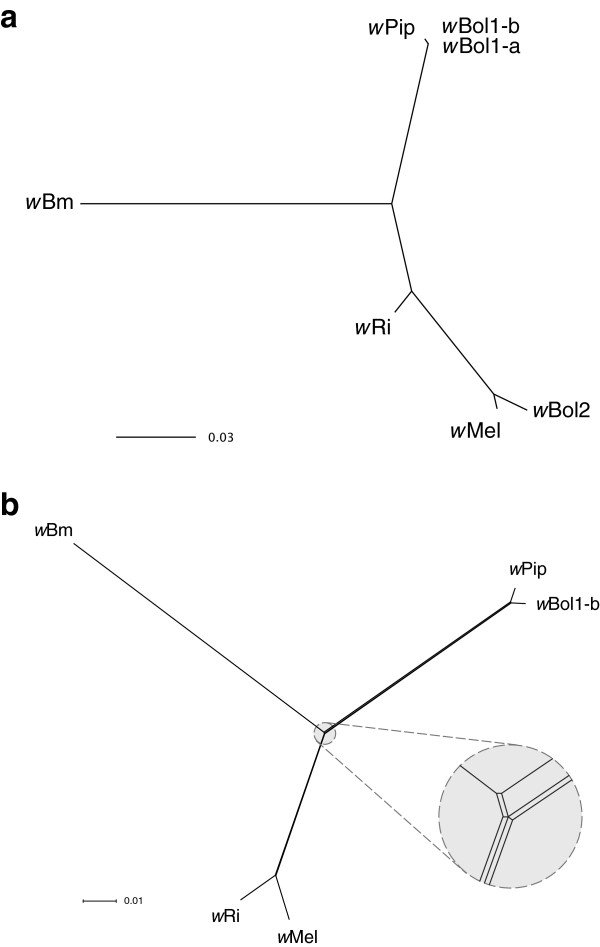
**(a)Maximum likelihood phylogeny, based on concatenated MLST genes,showing the relationships between the five *Wolbachia *strains for which genomes are available *(w*Mel, *w*Ri, *w*Pip, *w*Bm and *w*Bol1-b)and the two other strains known from *H.bolina,w*Bol1**-**a and *w*Bol2.** (**b**) A Neighbor-Net phylogenetic network based on a concatenated alignment of 654 core genes from the five complete genomes. Conflicting phylogenetic signals in the data are represented as boxes or parallelograms in the network. Very narrow boxes are present along the internal axes of the network, indicating that conflicting signal is present but minor in these genes. The large grey circle contains an expanded representation of the region of the network contained within the smaller grey circle, to make the narrow boxes visible.

In this paper, we addressed this problem by sequencing the genome of *w*Bol1-b from the butterfly *Hypolimnas bolina*. MLST phylogeny indicates that this strain is closely related to *w*Pip, the CI-inducing strain in the mosquito *Culex pipiens* (Figure 1a). Comparison of the two strains can therefore give insight into divergence over short periods of time associated with symbiosis in different host species, and with different phenotypes induced in those hosts. *w*Bol1-b induces male-killing, a phenotype observed in a range of insect symbionts, including several *Wolbachia* strains [17-20]. *w*Bol1-b is also notable for very high vertical transmission efficiency, leading to high prevalence [21] that affects the behaviour and ecology of the host [22]. This has driven the evolution of suppressor genes that prevent male killing from occurring in some host populations [23]; in these populations, *w*Bol1-b induces CI [24]. In addition to providing a view of *Wolbachia* divergence over short periods of time, the *w*Bol1-b genome also represents the second genome for a male-killing bacterium [25], and one for which interaction with the host can be investigated through examination of the suppression system.

This paper describes the annotated draft genome sequence of *w*Bol1-b, and compares its organization and gene content to those of four previously sequenced *Wolbachia* genomes. We identify and characterize genes that are specific to *w*Bol1-b, and show that a surprising number of these genes have been recently horizontally transferred from other taxa, including two genes that may be eukaryotic in origin. The results presented here provide a useful resource for future investigations of the genetic bases of male killing in *Wolbachia*, and further our understanding of the role played in *Wolbachia* genome evolution by frequent horizontal gene transfer.

## Results & discussion

### *Wolbachia* purification and genome sequencing

This is the first *Wolbachia* genome project to be completed using a combination of a novel method for preparing sequencing material and next-generation sequencing techniques. Because the ratio of *Wolbachia* to host DNA is typically very low, previous *Wolbachia* genome projects have required extensive and laborious purifications from extremely large numbers of host individuals before sequencing. Here we have overcome this difficulty in a number of ways. First, we used a short period of cell culture to amplify *Wolbachia* originally isolated from a single host individual. This removed the need for maintenance of extremely large numbers of butterfly hosts, and also increased the likelihood that our sequence data represent a single clonal lineage of *w*Bol1-b. As only a small number of cell culture passages were performed, it is unlikely that mutations could have arisen and fixed in the *w*Bol1-b genome during this process, so the sequence we obtained should represent that found in the insect. Secondly, we obtained *Wolbachia* from host cells using a version of a simple and rapid purification method. This method produces exceptionally pure endosymbiont material for sequencing: in our *w*Bol1-b 454 GS-FLX libraries fewer than 20% of sequence reads mapped to the host genome, while a refined version of the method can reduce this to fewer than 3% host reads [26]. Finally, the depth of sequence coverage obtained using next-generation sequencing (approximately 70X for this project) means that it is likely that a near-complete *Wolbachia* genome sequence could be obtained and assembled from a single sequencing run even if far more substantial host contamination were present. The combination of refined purification methods and development of sequencing technologies should facilitate the more rapid completion of future endosymbiont genome sequencing projects.

### Genome content

The draft genome of *w*Bol1-b contains 1,418,863 nucleotides (nt) in 13 scaffolds ranging in length from 3805 to 879,879 nt. This genome shares general characteristics with those of previously sequenced *Wolbachia* strains, including genome size, approximate number of coding sequences, and GC content (Table 1). The scaffolds contain 1257 predicted coding sequences. We used orthoMCL [27] to identify orthologous clusters of coding genes in the genomes of *w*Bol1-b, *w*Pip, *w*Mel, *w*Ri, and *w*Bm. We identified 654 core genes which are present in a single copy in each of the five strains, and a further 10 genes present in all strains but with a paralogous second copy (caused by a lineage-specific gene duplication) present in at least one strain (Additional file 1: Table S1). This is similar to the *Wolbachia* core genome size of 621 genes predicted by Ishmael et al. [16] by extrapolation from microarray-based comparative genome hybridization analyses of A-group *Wolbachia* strains.

**Table 1:** **General characteristics of *Wolbachia *genome sequences**

**Strain**	***w*Bol1**-**b scaffolds**	***w*Pip**	***w*Mel**	***w*Ri**	***w*Bm**
Supergroup	B	B	A	A	D
Genome size (nt)	1,418,863	1,482,455	1,267,782	1,445,873	1,080,084
G + C%	33.9	34.2	35.2	35.2	34.2
CDSs	1257	1386	1196	1150	903
tRNAs	34	34	34	34	34
rRNAs	1 each of 5S, 16S, 23S	1 each of 5S, 16S, 23S	1 each of 5S, 16S, 23S	1 each of 5S, 16S, 23S	1 each of 5S, 16S, 23S
ANKs	61	60	23	35	5
Genome accession #	CAOH01000001-CAOH01000144	NC_010981	NC_002978	NC_012416	NC_006833

ANKs are CDSs coding for one or more ankyrin repeat.

To assess the completeness of our assembly, we searched for orthologous gene clusters that were present in the other four sequenced *Wolbachia* genomes (and were thus potentially core genes) but that were absent from the *w*Bol1-b assembly. Only two clusters matched this pattern: a gene encoding an acetylornithine transaminase protein (WPa_0783 in *w*Pip), and a hypothetical protein-coding gene (WPa_0114). Orthologs of both of these genes were present in the *w*Bol1-b read data, but were on small contigs not assembled into scaffolds. We therefore believe that it is reasonable to assume that we have sequenced very close to 100% of the *w*Bol1-b genome, and that the vast majority of the non-repetitive protein-coding genes have been assembled and incorporated into scaffolds.

The genomes of the two B-group strains *w*Pip and *w*Bol1-b contain similar sets of genes likely to be involved in host interactions, including genes encoding membrane proteins and secretion systems. The *w*Bol1-b genome contains the 14 genes coding for the proteins that make up the *Wolbachia* type four secretion system (T4SS) [10,28]. As in the four other fully sequenced *Wolbachia* strains, 11 of the *w*Bol1-b T4SS genes are grouped into two operons (the first including *virB4*, *virB3* and four copies of *virB6*, and the second *virB8*, *virB9*, *virB10*, *virB11* and *virD4*), while the second copies of *virB4*, *virB8* and *virB9* are distributed elsewhere in the genome.

Ankyrin (ANK) repeat domains are abundant in *Wolbachia* genomes [10,29,30], and may be involved in interacting with or manipulating the host e.g. [31,32]. A total of 61 ANK coding genes were found in the *w*Bol1-b draft genome, one more ANK coding gene than in *w*Pip [30], and substantially more than the two A group strains previously sequenced, *w*Mel and *w*Ri, which have 23 and 35 ANK genes respectively (Table 1). Despite the similar number of ANK genes in *w*Pip and *w*Bol1-b, not all are orthologous between the two strains: eight of the *w*Bol1-b ANK genes were not grouped into ortholog clusters with *w*Pip members. Several of these genes have been lost or pseudogenised in the *w*Pip lineage after divergence from *w*Bol1-b, while others appear to have been newly introduced into the *w*Bol1-b genome.

### Genome recombination and rearrangement

MLST data had previously indicated that *w*Bol1-b and *w*Pip were closely related (Figure 1a). A phylogenetic network analysis (Figure 1b) based on the concatenated alignments of the 654 core genes from the five genomes confirms that these are the most closely related strains sequenced to date. Boxes or parallelograms in the network indicate conflicting phylogenetic signal [33]; this network contains only very narrow boxes, indicating that there is a low level of conflict in the data at the nucleotide level. This is perhaps somewhat unexpected, given the high levels of recombination previously reported to occur between *Wolbachia* genomes e.g. [11], but there is a simple explanation: it is likely that most homologous recombination occurs between closely related strains, and thus within supergroups. As we have data from only one or two strains per supergroup, this network is unlikely to capture the signal of the majority of homologous recombination that may be occurring between *Wolbachia* genomes. Our network does suggest, however, that homologous recombination of core genes between supergroups is not rampant.

**Figure 2:**
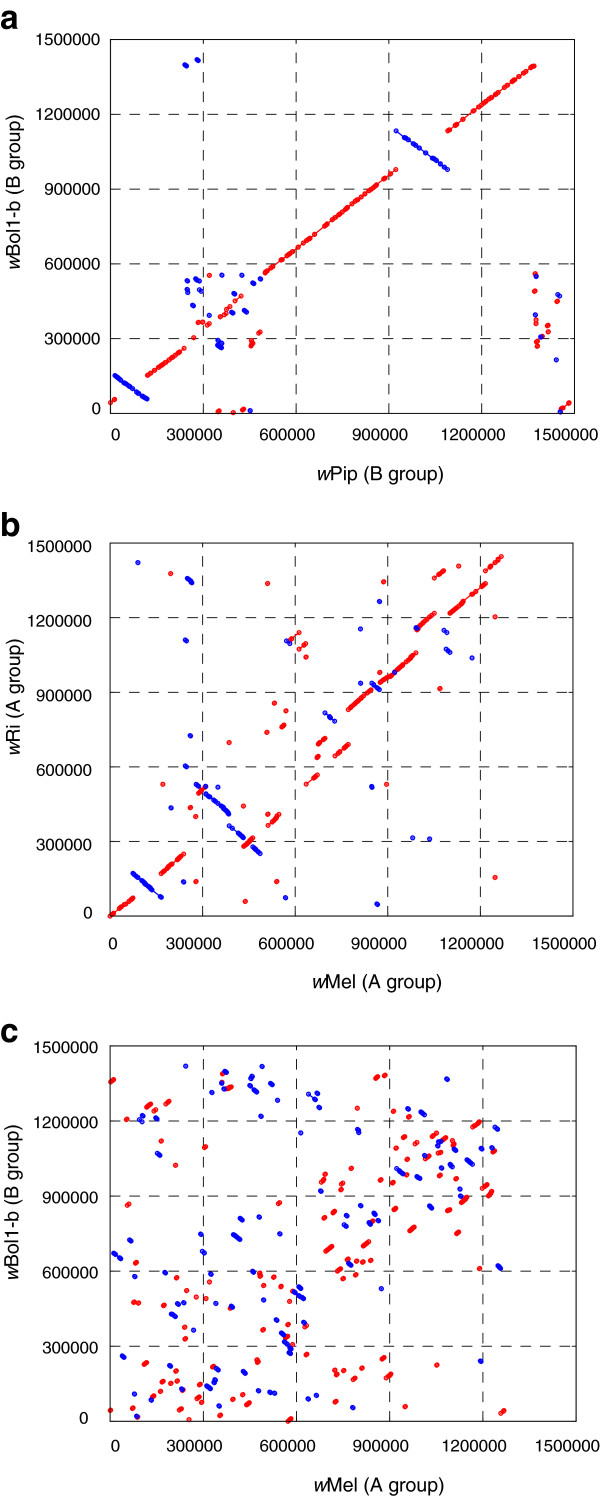
**Dot plots showing differences in syntenic conservation between *Wolbachia *genomes.** Dots and lines represent unique genomic sequence matches, red for a forward match and blue for a reverse match (inversions). Comparisons are shown between the genomes of (**a**) *w*Bol1-b and *w*Pip, the two complete B-group genomes, (**b**) *w*Mel and *w*Ri, the two complete A-group genomes, and (**c**) *w*Bol1-b and *w*Mel, a representative comparison between A- and B-group genomes. Numbers along the axes represent genome coordinates.

Previous comparative analyses of *w*Mel, *w*Ri, *w*Pip and *w*Bm have shown a relatively high level of genome rearrangement between these four *Wolbachia* strains [12,13]. In contrast, large regions of the *w*Bol1-b genome are almost perfectly colinear with their corresponding regions in *w*Pip (Figure 2). Scaffolds 2 and 20 cover approximately 82% of the *w*Bol1-b genome, and show high syntenic conservation with *w*Pip, with the notable exceptions of one large inversion in each scaffold. The largest inversion in scaffold02 is approximately 156,400 nt long and contains 171 coding sequences (CDSs, from wBol1_0654 to wBol1_0835), while the largest inversion in scaffold20 is approximately 93,000 nt and contains 82 CDSs (from wBol1_1153 to wBol1_1242).

*Wolbachia* genomes are extremely rich in insertion sequences (IS) with transpositional activity [34]. These elements are often associated with junctions between syntenic blocks [11,35]. Three of the four junctions of the two major inversions described above are associated with a copy of an IS2 transposase in *w*Pip (WPa_0116, WPa_0846 and WPa_1024). This transposase is present in 46 annotated copies in the *w*Pip genome, 44 of them identical. The *w*Bol1-b assembly contains one degenerate copy of this transposase, and there are at least two other moderately diverged but complete copies present in short contigs not included in the assembly. The lack of sequence divergence between the different copies of this transposase gene in *w*Pip suggests that it may be active and involved in ongoing transpositional activity in both *w*Bol1-b and *w*Pip.

### WO prophage and *w*Bol1-b-specific genes

Each of the previously sequenced A- and B-group *Wolbachia* genomes contains between two and five prophage *WO* copies. The draft *w*Bol1-b assembly currently contains nine fragmentary prophage regions, many of which are short and associated with the end of scaffolds. This suggests that these regions are incompletely assembled prophages, and that the true number of prophage copies in the genome is substantially smaller. The longest prophage region, and probably the only one completely assembled, consists of the complete length of scaffold 17, from wBol1_0161 to wBol1_0219. This region is almost precisely colinear with the *w*Pip prophage WOPip5, as defined in [36]. Colinearity is disrupted only by a small number of insertion or deletion events, each affecting between one and five genes. The full list of putative prophage regions in the assembly is given in Additional file 2: Table S3.

Many horizontal gene transfer events into *Wolbachia* are likely to be mediated by bacteriophage, which are known to transfer laterally between *Wolbachia* strains coinfecting the same host [37] and are capable of transferring flanking non-phage genes in the process [38], thus facilitating horizontal gene transfer and genome diversification. To identify possible recent horizontal gene transfers into the *w*Bol1-b genome, we used *w*Bol1-b genes that had not been clustered with any other gene in the orthoMCL analysis (and were therefore putatively *w*Bol1-b-specific) as blastp queries against the NR database. 26 of these genes had blastp matches to genes from other *Wolbachia* strains not included in the clustering analysis, and are thus components of the accessory genome but are not *w*Bol1-b-specific. A total of 44 genes are present in wBol1-b but no other currently sequenced *Wolbachia* strain (Additional file 2: Table S4). Of these, 35 had no NR matches; these may be very rapidly evolving genes, genes in the late stages of degeneration, or the result of horizontal transfer from a genome not yet represented in the database [39]. It is also possible that some, especially the shorter of these genes, could be artefacts of the annotation process. Finally, nine *w*Bol1-b-specific genes lacked *Wolbachia* homologs but had high-quality matches to non-*Wolbachia* genes in the NR database. All but one of these genes are either within or adjacent to phage regions. We searched for degenerate or unannotated copies of these genes in the *w*Pip genome and found no evidence of them, and it is likely that they represent recent phage-mediated horizontal gene transfers into the *w*Bol1-b genome that occurred subsequent to divergence from *w*Pip. These genes and their homologs are described below.

Two contiguous genes, wBol1_0262 and wBol1_0265, encode proteins with radical SAM (*S*-adenosylmethionine) domains, which are known to play diverse molecular roles, including interaction between intracellular bacteria and their hosts [40,41]. These genes are divergent homologs of two contiguous genes in the genome of the Actinobacterium *Micromonospora aurantiaca*. The transposase gene wBol1_0093 has homologs in a diverse range of bacterial taxa, including the sponge symbiont Rhodobacteraceae bacterium KLH11 and the plant-associated environmental bacteria *Burkholdaria cenocepacia* and *Dyadobacter fermentans*, but there is insufficient phylogenetic resolution to determine which of these is most closely related to wBol1_0093 (Additional file 2: Figure S2a). wBol1_0035, which encodes a hypothetical protein, clusters with genes from the cyanobacterium *Synechococcus* sp CC9311 and the pathogen *Legionella longbeachae* (Additional file 2: Figure S2b), though with relatively low bootstrap support. wBol1_0187, also encoding a hypothetical protein, clusters with 100% bootstrap support with a gene from the methanogenic archaeon *Methanococcoides burtonii* (Additional file 2: Figure S2c). Divergent homologs of this gene are annotated in the genomes of A group strains, but these are distant from wBol1_0187 in the phylogenetic tree and form a separate, strongly supported clade with a gene from the homoacetogenic bacterium *Clostridium ljungdahlii*, suggesting two independent transfers into *Wolbachia* genomes.

The origin of two other contiguous *w*Bol1-b-specific genes, wBol1_0256 and wBol1_0257, appears more complex. Both genes have as their top NR blastp match the *Solenopsis invicta* (fire ant) gene SINV_00084. The 5’ and 3’ ends of SINV_00084 are similar to fragments of insect Golgi SNAP receptor complex genes, while the highly internally repetitive central portion of the gene matches a region of the *Rickettsia massiliae dnaE2* gene, RMA_0751, which is part of the *tra* cluster region [42]. This gene cluster, which encodes proteins involved in conjugal DNA transfer, is thought to have been laterally transferred into *Rickettsia* from *Protochlamydia amoebophila*, an obligate symbiont of amoebae [43]. Given the highly repetitive nature of the sequences involved, and the few taxa for which matching sequences are available in Genbank, it is not currently possible to determine the evolutionary history of the possible transfers of this genetic region between *w*Bol1-b, *R*. *massiliae*, *S*. *invicta* or some number of possible intermediates not yet represented in the database. Finally, two additional *w*Bol1-b genes, wBol1_1091 and wBol1_1092, discussed in further detail below, may have originated in eukaryotes.

What are the proximate sources of these horizontally transferred genes? There is clearly no single taxonomic group represented in the NR database that shares close homologs of this complete set of genes with *w*Bol1-b. Furthermore, for several of these genes, the closest known homologs are moderately divergent from the copies in *w*Bol1-b, and it is unlikely that the genetic transfer took place directly between these taxa and *Wolbachia*. These genes are found associated with different phage regions of the *w*Bol1-b genome, and may be the result of multiple independent transfers since divergence from *w*Pip. Together, these observations suggest that the introduction of new genes into *Wolbachia* genomes is ongoing and frequent, and that the phage that mediate these transfers carry genetic material from an exceptionally diverse group of organisms.

### Horizontal gene transfer between *w*Bol1-b and eukaryotes

Two *w*Bol1-b-specific genes that each contain a secA domain may have been transferred from eukaryotic rather than prokaryotic taxa. Proteins containing secA domains are best characterized in bacteria, where they act as ATPases mediating translocation of preproteins through the cytoplasmic membrane [44]. The core *Wolbachia* genome includes a copy of a typical bacterial secA (encoded by WD0549/WRi_003630/WPa_0368/wBol1_0067/Wbm0266) that is of unexceptional length for *Wolbachia* genes (2604 nt) and has orthologs in closely related α-Proteobacterial genera *Ehrlichia* and *Anaplasma*. The secA genes wBol1_1091 and wBol1_1092, however, show a different pattern. These two genes are unusually long for *Wolbachia* genes: wBol1_1091 is 4488 nt in length and wBol1_1092 is 11,829 nt. Both have full-length matches in the NR database only to insect proteins.

wBol1_1091 has multiple full-length matches to hypothetical proteins in *Culex quinquefasciatus* and *Aedes aegypti*. The 5’ half of the gene has matches only to the mosquito genes, but the 3’ half of the gene, which contains the secA domain, also has further hits to basal eukaryotes, including *Polysphondylium pallidum*, *Tetrahymena thermophila* and *Salpingoeca* sp. There are no close bacterial matches. The highest-ranked bacterial match has low-level and fragmentary sequence similarity with a portion of the secA domain region of wBol1_1091, and has a bit score of only 108, compared to scores of 200 to 957 for the eukaryotic matches.

wBol1_1092 has full-length hits to multiple secA proteins in the jumping ant *Harpegnathos saltator*, and a near-full length hit to a hypothetical protein in *Daphnia pulex*. The region of the protein containing the secA domain has hits to hypothetical proteins in other insects including *Drosophila willistoni*, *Culex quinquefasciatus* and *Tribolium castaneum*. This region also has less significant hits to proteins from basal eukaryotes including *Tetrahymena* and *Polysphondylium*, and more distantly to bacterial secA proteins.

**Figure 3:**
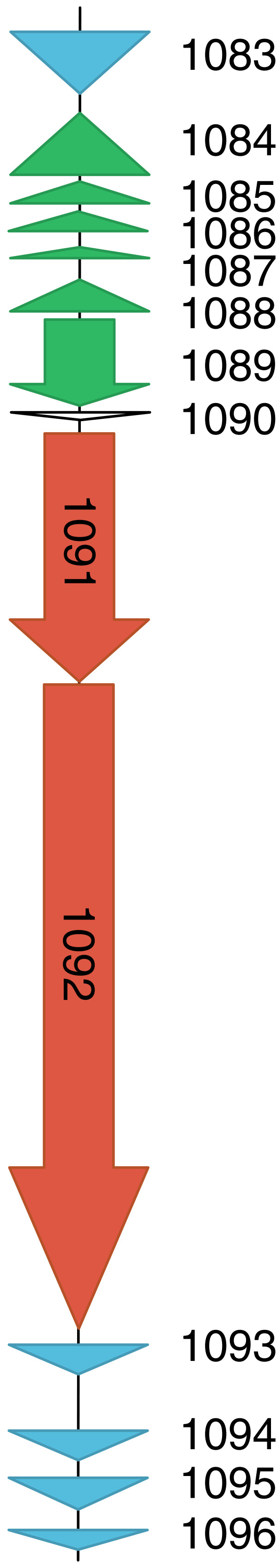
**Location of horizontally transferred secA genes**, **wBol1**_**1091 and wBol1**_**1092,in the *w*Bol1**-**b genome.** Arrows represent CDSs, are drawn to scale, and are labelled with abbreviated locus names (e.g. wBol1_1091 is 1091). Core *Wolbachia* genes are shown in green, non-core genes with identifiable *Wolbachia* homologs are in blue.

In the *w*Bol1-b draft genome assembly, wBol1_1091 and wBol1_1092 are surrounded by known *Wolbachia* genes (Figure 3). We confirmed the position of these eukaryotic-like secA genes in the *Wolbachia* genome, and their absence from uninfected host material, using PCRs spanning wBol1_1089-wBol1_1091 and wBol1_1092-wBol1_1093, on infected and antibiotic-treated lines of *H*. *bolina*.

Other *Wolbachia* homologs of these secA genes were not found in complete genomes or in the NR database. However, matches were found to whole genome shotgun sequence data from the NC48S strain of *Drosophila simulans*, sequenced as part of the *D*. *simulans* genome project [45], when blasting against the NCBI WGS database. The NC48S line was collected in Noumea in 1991, and is superinfected with two *Wolbachia* strains, A group *w*Ha and B group *w*No [46]. None of the other six lines of *D*. *simulans* sequenced for the *D*. *simulans* genome project, whether *Wolbachia*-uninfected or infected with *w*Ri, appeared to contain sequence matches to these secA genes. To determine which of *w*Ha and *w*No carries the secA homologs, we used NC48S lines in which the superinfection has been separated out into single infections. We amplified and sequenced portions of the homologs of both wBol1_1091 (516 nt) and wBol1_1092 (3018 nt) from the line infected with *w*Ha, but could not amplify these regions in either the line infected with *w*No or the antibiotic-treated uninfected lines. The sequences from *w*Bol1-b and *w*Ha were 93.4% (wBol1_1091) and 94.6% (wBol1_1092) identical at the nucleotide level. The secA genes from *w*Bol1-b and *w*Ha cluster together phylogenetically to the exclusion of their insect homologs (Additional file 2: Figure S3). wBol1_1092 is evolving under purifying selection in these two strains (ω = 0.266; ω < 1 with *P* < 0.001), suggesting that it is functional. wBol1_1091, however, appears to be evolving neutrally (ω = 0.945; ω not significantly < 1).

**Figure 4:**
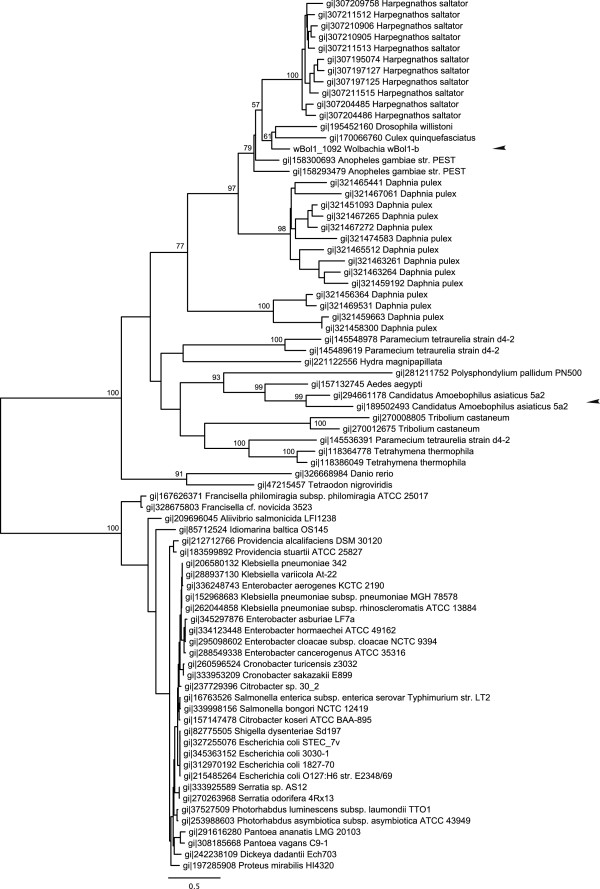
**Maximum likelihood phylogenetic tree based on the amino acid sequences of *Wolbachia *secA gene wBol1**_**1092 and its homologs.** Prokaryote and eukaryote secA genes cluster into two separate, well-supported clades. There are two apparent transfers from eukaryote to prokaryote species, marked with arrowheads. Bootstrap values over 50 are shown; for clarity they have been removed from short internal branches within the prokaryote clade and the large *Daphnia* and *Harpegnathos* clades. Each tip is labelled with the sequence’s Genbank GI number and the species name.

In what direction did the horizontal transfer of these genes between *Wolbachia* and insects occur? The taxonomic distribution of secA domain proteins in eukaryotes makes it clear that they have been susceptible to horizontal transfer between lineages. However, blastp searches using wBol1_1091 or wBol1_1092 as queries reveal a clear delineation between the full-length, high-similarity eukaryotic matches and lower-quality matches to bacteria, a pattern that is confirmed by phylogenetic analyses. The eukaryotic homologs of wBol1_1092 form a strongly supported clade separate from the prokaryotic homologs (Figure 4). Within the eukaryotic clade, there have been two transfers of this gene into bacteria: one represented by wBol1_1092, and the other by two *Candidatus* Amoebophilus asiaticus genes, Aasi_1610 and Aasi_1144, which cluster with an *Aedes aegypti* gene. Unlike wBol1_1092, however, these *Candidatus* Amoebophilus genes do not have full-length matches to insect secA proteins, with sequence similarity only in the secA domain.

The presence of introns in genes subject to inter-domain horizontal gene transfer is often taken as evidence that transfer occurred from eukaryote to prokaryote. Although many of the eukaryotic secA genes are annotated with introns, most of these introns appear to be spurious (Additional file 2). There is little evidence of true introns in the eukaryotic secA genes most closely related to the *Wolbachia* genes, but this does not indicate that the transfer took place in the opposite direction: a substantial proportion of genes in these eukaryotic genomes are intronless, and there is no reason to believe that they all represent transfers from prokaryotes.

The extremely long gene length and phylogenetic analyses suggest that transfer of these genes occurred from eukaryotes into the *Wolbachia* genome. This matches the pattern of evidence observed for the gene WD0513, which was transferred between mosquito taxa and *w*Mel/*w*Pip [47,48], and raises the possibility that *Wolbachia* genomes are able to receive, harbour, transfer, and possibly use protein coding genes of eukaryotic origin.

### Possible genomic bases of male killing

Although male-killing bacteria have been described from diverse taxa, including *Wolbachia*, *Spiroplasma*, *Rickettsia* and *Arsenophonous*[17-20], little is known about the mechanism of male-killing in any of these systems. *Spiroplasma poulsonii*, the best characterized example to date, kills *Drosophila melanogaster* males only in the presence of all five peptides of the host dosage compensation complex [49], and this killing occurs during a narrow developmental period early in embryogenesis [50]. It has been speculated that male-killing in this system may occur through uncoordinated expression of the apoptosis pathway across the embryo, but the precise mechanism and the molecules secreted by *Spiroplasma* to initiate it are both unknown [50]. In contrast, *Arsenophonous* kills males in *Nasonia* by inhibiting the formation of maternally-inherited centrosomes, which are required for early male development [51]. The mechanisms of male-killing caused by strains of *Wolbachia* are generally less well characterized, and even more varied. Male-killing *Wolbachia* in *D*. *bifasciata* cause severe defects in chromatin remodelling and spindle organization in male embryos, leading to developmental failure [52]. The *Wolbachia* strain *w*Sca feminizes males of its host, the moth *Ostrinia scapulalis*, and it is thought that it is a mismatch between the genetic and phenotypic sexes that causes male death [53]. The mechanism underlying male-killing by *w*Bol1-b in *H*. *bolina* is uncharacterized, but it can act in both well-developed embryos and young larvae, suggesting that it is not due to specific targeting of an early developmental pathway in the host [54].

The diversity of mechanisms known to underlie male-killing in different systems makes it difficult to predict *a priori* what the physiological or genetic basis of this phenotype is in *w*Bol1-b. For this reason, using comparative genomics to identify candidate genes involved in male-killing is a valuable complementary approach to this question. Genes present in *w*Bol1-b but absent from the genome of the closely related non-male-killing strain *w*Pip are initial candidates for investigation. We have identified a number of genes specific to this genome (Additional file 2: Table S4), including functionally annotated single-copy genes, as well as genes coding for hypothetical proteins and paralogs of known *Wolbachia* genes, and these should be targets for further research.

It is also possible that the expression of male killing is mediated by additional factors, such as changes in the tropism or level of expression of genes that are also present in non-male-killing strains of *Wolbachia*. The availability of the *w*Bol1-b genome will facilitate future transcriptomic analyses of expression patterns. Male killing could also potentially arise as a result of changes in gene sequence of common genes leading to modifications of their function or expression. We performed branch-specific and branch-site analyses of positive selection [55] to test for adaptive evolution in core genes along the lineage leading to *w*Bol1-b, but there was insufficient power to identify significant changes in selection due to the short length of this branch of the phylogeny (data not shown). Finally, there are a number of studies showing that, for at least some strain-host combinations, the expression of male killing is host-dependent and that some hosts are able to evolve to repress male killing [56-58], and this should be taken into account in future investigations of the mechanism underlying this phenotype.

## Materials & methods

### *w*Bol1-b origin and transfer into cell culture

A *w*Bol1-b-infected butterfly (G0) was collected on the island of Moorea (French Polynesia) in February 2006. We dissected out the abdomen of a mature G1 *H*. *bolina* under aseptic conditions. The abdomen was surface-sterilized in 70% ethanol, then incised in a small volume of 96% ethanol. The eggs extracted from the abdomen were briefly surface-sterilized in 70% ethanol. Approximately ten butterfly eggs were transferred into a 1.5 mL centrifuge tube and washed three times with 1 mL of a sucrose-phosphate-glutamate solution (SPG) [59], which briefly maintains *Wolbachia* viability outside host cells. Eggs were re-suspended in 1 mL SPG and crushed against the centrifuge tube walls using a micropestle.

Each well of a vial plate was filled sequentially with 200 μL monolayers of *Aedes albopictus RML12* cells (80% confluent), 1 mL of Mitsuhashi and Maramorosh insect medium [60] and 500 μL of the egg extract. The plate was centrifuged at 800×g for 1 hour, then incubated overnight at 26°C. Cells were re-suspended and added to 5 mL of fresh insect cell culture medium in a 20 cm^2^ flask, and then maintained *invitro* by transfer into fresh medium as per normal every four days [61].

### *Wolbachia* DNA preparation

We grew wBol1-b-infected cells for 6–15 weeks after the transfer of the infection to *RML12* cell culture. From a total of 500 cell culture flasks (175 cm^2^), six *w*Bol1-b DNA samples were purified using the protocol described by [26], followed by an extra separation step on percoll gradient. Six Beckman ultra-clear centrifuge tubes (9/16 × 3 ½ inch, 14 × 89 mm), each containing four density layers (from bottom to top: 2 mL of 60% Percoll, 4 mL of 40% Percoll, 3 mL of 20% Percoll and 2.7 mL of 10% Percoll) were prepared on ice. The gradients were loaded with 700 μL sample and placed in the swinging SW41 rotor buckets of a Beckman Optimal-L-80 XP ultracentrifuge and centrifuged for one hour at 10,900xg at 4°C. This step separates *Wolbachia* cells from other cell components and debris based on their density, and four opaque bands of cellular material appeared between the different Percoll layers. The bottom opaque band (band #4) between the 40% and 60% Percoll layers was collected.

DNA from G0 butterfly, G1 butterfly and transinfected cells was extracted using a Qiagen DNAeasy blood and tissue extraction kit. The *w*Bol1-b infection status of the samples was confirmed by PCR with *w*Bol1-b specific primers 81 F/522R and Gp1bF/R [15] respectively, Additional file 2: Table S2, [62]. The DNA composition of band #4 of the Percoll density gradient was also characterized by PCR, after extracting the DNA from an aliquot using phenol-chloroform. Presence of *Wolbachia* strain *w*Bol1-b DNA was confirmed by PCR amplification of the surface protein gene *wspb*, using primers 81 F/522R, and the ankyrin gene WD637, using primers 693 F/693R. Primer pairs for the *Aedes aegypti* 18S rRNA gene and protein-coding genes RPS7 and EF (18S-F/18S-R, AgRPS7-F/AgRPS-R and EF-F/EF-R) amplified *Aedes albopictus* contaminating DNA. The12S rRNA gene was used as a mitochondrial DNA marker. The purification quality was assessed by running 1 μg of the Wolbachia DNA in a 1% agarose gel for 40 min, with 1 kb DNA ladder.

### *w*Bol1-b genome sequencing and assembly

Approximately 5 μg of *Wolbachia* DNA was provided to AGRF (Australian Genome Research Facility), which generated a 454 GS-FLX shotgun library. An additional sample was then provided to AGRF to generate a 454 GS-FLX paired-ends library. Libraries were combined for genome assembly.

Genome assembly was done by AGRF following the protocol provided by the platform supplier (Roche) and based on a shotgun-data-first addition order. Contaminating mosquito sequence was identified by using contigs as blastN queries against the NR database and identifying high-similarity matches. The assembly was manually edited using Artemis [63]. The final assembly consists of 91 contigs, ranging from 644 bp to 155817 bp in length, arranged into 13 scaffolds, and an additional 53 unscaffolded contigs with lengths less than 2 kb. The sequence data has been deposited at the European Nucleotide Archive (CAOH01000001-CAOH01000144) in BioProject PRJEB566.

We confirmed that the strain sequenced was a clonal lineage by mapping the reads to the assembly using Newbler v2.6 (Roche) and checking for evidence of well-supported SNPs, which would suggest that a mixed culture had been sequenced. All high-confidence SNPs were associated with known imperfect repeats (data not shown), indicating that the strain sequenced is highly likely to have been clonal.

### Annotation

Initial annotation of the *w*Bol1-b draft genome assembly was performed using SUGAR (Simple Unfinished Genome Annotation Resource), an annotation pipeline consisting of several Perl scripts, controlled by a user defined instruction file (Szubert & Beatson, In Prep.). The program makes use of the NUCmer component of the MUMmer 3.0 package [64] for ordering an unfinished genome against at least one reference sequence. Glimmer 3.02 [65] was used for protein coding gene calling (after punctuating contig boundaries with a six frame stop-start sequence), based on a set of observed long ORFs, with optional scanning for genes matching over boundaries, and improvements to paired ends derived scaffolding. Automated annotation of proteins was based on a diminishing identity threshold scale for Blastp [66] matches against protein databases consisting of (1) the reference genome *w*Pip, (2) other *Wolbachia* genomes, (3) swiss-prot and (4) the non-redundant database (NR). Annotations based on profile matches in Pfam [67], TIGRFAM [68] and COG [69] databases were also supplied. t-RNA genes were predicted using TE-SCAN [70].

### Ortholog prediction

We performed an all-versus-all blastp analysis of the predicted proteomes of *w*Mel, *w*Ri, *w*Pip, *w*Bol1-b and *w*Bm, then used orthoMCL [27] to group orthologs and their recent paralogs (lineage-specific duplications) into ‘ortholog clusters’. We ran the analysis multiple times using E-value cut-offs of 1e-05 and 1e-10, and with inflation values of 1.2, 1.5, 2.5 and 5. The great majority of core ortholog clusters were identical across runs; we performed all further analyses on results of the run with default settings (E-value 1e-5, inflation 1.5). We used purpose-written Perl scripts to parse the results of this analysis and identify clusters of core genes. To confirm that the ortholog clustering was reasonable, we compared the core gene ortholog sets produced by orthoMCL to those predicted using two other methods: a simple all-versus-all mutual best blast hit analysis, and the sets of single-copy positional homologs inferred by Mauve [71] after alignment of the five genome sequences. 659 core ortholog sets were predicted by at least one of the three methods. Of these, 577 (88%) were predicted by all three, 67 (10%) by two, and 15 (2%) by one method. For the 67 ortholog sets predicted by two methods, 65 of them were predicted by orthoMCL and mutual best blast hits, but not by Mauve. Many of the ortholog sets not called by Mauve had substantial differences in gene length between orthologs, or synteny breaks adjacent to one or more orthologs in the genome alignment, which were generally sufficient to explain the difference in predictions. We manually inspected all ortholog sets predicted by one method only, and approximately 50% of the sets predicted by two methods. In almost all cases, the orthoMCL prediction was supported by inspection.

### *w*Bol1-b-specific genes

To identify genes specific to *w*Bol1-b, we used the amino acid sequences of all of the *w*Bol1-b genes that were not clustered with any other genes in the orthoMCL analysis as blastp queries against the NR database with a very low stringency E-value threshold of 10. Genes were considered putatively wBol1-b-specific if they had either no hits to the NR database with this cut-off, or had no hit to any *Wolbachia* gene with a better E-value than the best hit to a non-*Wolbachia* gene.

### Phylogenetic analyses

For the MLST tree, we manually aligned nucleotide sequences of the *coxA*, *fbpA*, *ftsZ*, *gatB* and *hcpA* genes [72] from *w*Mel, *w*Ri, *w*Pip, *w*Bol1-a, *w*Bol1-b, *w*Bol2 and *w*Bm, obtained from Genbank, then concatenated the alignments. We inferred a phylogenetic tree using PHYML [73], using the HKY substitution matrix, a discrete gamma model with four rate classes and a gamma shape parameter estimated from the data. For the phylogenetic network, we used t_coffee [74] to align sequences of the 654 core genes from the five *Wolbachia* genomes. These single-gene alignments were concatenated to form an alignment 681,717 nt in length. A Neighbor-Net network [33] was inferred from this alignment using default parameter values in SplitsTree [75]. For the phylogenetic analyses of secA and other putatively horizontally transferred genes, we aligned amino acid sequences using t_coffee [74], then edited and trimmed alignments by eye. We inferred phylogenetic trees using PHYML [73], using the JTT substitution matrix and four substitution rate classes with the gamma parameter estimated from the data.

### Synteny analysis

Synteny between the *w*Bol1-b assembly and other complete *Wolbachia* genomes was visualised using NUCmer (with parameter settings --maxgap = 500, --mincluster = 100) and mummerplot [64].

### *w*Bol1-b *WO* prophage regions

Putative prophage regions were identified using a combination of two methods. First, the *w*Pip, *w*CauB and *w*VitB *WO* prophage proteins (as defined in [36]) were used as blastp queries against the *w*Bol1-b protein sequences. Secondly, we identified *w*Bol1-b genes that clustered with *w*Pip prophage genes in the orthoMCL results. Boundaries of the putative prophage regions were determined by a combination of checking homology with *w*Pip and *w*CauB prophages and manual assessment of gene annotations in boundary regions.

### Sequencing of secA genes

The presence and position of secA genes in *w*Bol1-b was confirmed by sequencing PCR products obtained using primers spanning the wBol1-b_1089-wBol1-b_1091 boundary (mutLSecA1-F + mutLSecA1-R) and the wBol1-b_1092-wBol1-b_1093 boundary (SecA2Tran-F + SecA2 Tran-R). The wBol1-b_1092 ortholog in wHa was amplified using various combinations of primers 1092–2, -3, -4, -5, -6, which were designed based on the *w*Ha sequence fragments present in the NCBI WGS database. The wBol1-b_1091 *w*Ha ortholog was amplified using primers 1091-F and 1091-R, also based on *w*Ha sequence. Primer sequences are listed in Additional file 2: Table S2. PCR cycling conditions were as follows: 94°C 3 min, (94°C 30 s, 52°C 30 s, 68°C 150 s) × 35 cycles, then 68°C 10 min. The reaction mixture contained 625 nM of each primer, 125 μM dNTPs, 1.5 mM MgSO_4_, 20 ng of DNA and 0.5 μL of proof-reading Elongase enzyme mix (Invitrogen) in a final volume of 25 μl. PCR products were separated in 1% agarose gels and stained with ethidium bromide. Purified PCR products were sequenced at the Micromon facility (Monash University, Clayton, Australia) to confirm the insertion into the *Wolbachia* genome and the identity of the sequences.

### Selection analyses

To test whether genes were evolving under purifying selection, we used codeml [55]. We used likelihood ratio tests (LRT) first to confirm that there was no significant variation in ω values between lineages, and then to compare the likelihood of a model of evolution that fixed ω = 1 with that of a model that allowed ω to be estimated from the data. If the latter LRT was significant, we checked that the estimated ω was < 1.

## Competing interests

The authors declare that they have no competing interests.

## Authors’ contributions

SO, AD, MW, IIO, JB, EM, GH and SC participated in the design and oversight of the study. AD, IIO and CM carried out laboratory work. SB, JS, AD and MW annotated the genome. AD and MW analysed the data and drafted the manuscript. All authors contributed to and approved the final manuscript.

## Supplementary Material

Additional file 1 **Table S1.** Clusters.xls, which lists ortholog clusters of core genes as identified by orthoMCL. Click here for file

Additional file 2 **Figure S1.** Establishment of cell culture; **Table S2.** primers used in this study; **Table S3.** partial prophage regions in *w*Bol1-b; **Table S4.***w*Bol1-b-specific genes; **Figure S2.** phylogenetic trees of horizontally transferred genes; **Figure S3.** phylogenetic tree of wBol1_1092 including partial *w*Ha homolog; Introns in eukaryotic secA genes; Note on annotation of WD1302. Click here for file
