# Supplementary material for: Draft genome sequence of the male-killing Wolbachia strain wBol1 reveals recent horizontal gene transfers from diverse sources
Source: BMC Genomics. 2013 Jan 16;14:20. doi: 10.1186/1471-2164-14-20 (PMC3639933; doi:10.1186/1471-2164-14-20)
Supplement: Additional file 2 — Figure S1. Establishment of cell culture; Table S2. primers used in this study; Table S3. partial prophage regions in wBol1-b; Table S4.wBol1-b-specific genes; Figure S2. phylogenetic trees of horizontally transferred genes; Figure S3. phylogenetic tree of wBol1_1092 including partial wHa homolog; Introns in eukaryotic secA genes; Note on annotation of WD1302. [file 1471-2164-14-20-S2.docx]

**Supplementary Information**

Supplementary information for this paper consists of two files:

(1) Supplementary Table 1, clusters.xls, which lists ortholog clusters of core genes as identified by orthoMCL.

(2) This document, which consists of the following sections:

| **Section** | **Page** |
| --- | --- |
| Purification of *Wolbachia* from cell lines, including Fig S1 | 2 |
| Table S2, primers used in this study | 4 |
| Table S3, partial prophage regions in the wBol1-b assembly | 5 |
| Table S4, *w*Bol1-b-specific genes | 9 |
| Fig S2a, phylogenetic tree of wBol1_0093 and homologs (HGT) | 11 |
| Fig S2b, phylogenetic tree of wBol1_0035 and homologs (HGT) | 12 |
| Fig S2c, phylogenetic tree of wBol1_0187 and homologs (HGT) | 13 |
| Fig S3, phylogenetic tree of the horizontally transferred secA gene wBol1_1092 including partial wHa ortholog | 14 |
| Introns in eukaryotic secA genes | 15 |
| Note on the annotation of WD1302 | 16 |
| References for Supplementary information | 17 |

***w*Bol1-b establishment in cell lines and purification for sequencing***: w*Bol1-b was purified from the abdomen of an infected female *Hypolimnas* *bolina* butterfly, collected in Moorea, French Polynesia. *Wolbachia* was established in cell culture and maintained with serial passage until sufficient material was purified for sequencing. Figure S1 outlines the purification process, in particular the Percoll gradient and the characterization of the different bands of dense material that form in the interphase between the layers.

**Fluorescence in situ hybridization:** FISH was performed on confluent RML12 cell lines following the protocol described by [1], but using the w1 (5’-AATCCGGCCGARCCGACCC-3’) and w2 (5’-CTTCTGTGAGTACCGTCATTATC-3’) rhodamine-labelled probes described by [2].

**Figure S1** Overview of the establishment of *w*Bol1-b in cell culture and the purification of *Wolbachia* DNA for sequencing. (A) Female *H. bolina*. The photo is courtesy of Marten Runsquit and is from an orange fermale form from Tonga. Purified *w*Bol1-b *Wolbachia* extracted from the abdomen of a mature female butterfly was used to infect RML12 *A. albopictus* cell lines. *Wolbachia* numbers were amplified following serial passage in cell lines. (B) Fluorescence in situ hybridization (FISH) showing the presence of *w*Bol1-b *Wolbachia* inside the cytoplasm of RML12 infected cells. *Wolbachia* is stained in red with rhodamine-labelled specific probes. DNA is stained in blue with DAPI. (C) Characterization of the four bands obtained after *w*Bol1-b purification by Percoll density gradient. Four opaque bands (Bands #1 - 4) appear at the top limit of each of the four layers. The four bands were characterized by PCR to determine the presence of *Wolbachia* and host DNA. Band 4 contains the highest Wolbachia to host DNA ratio and was collected to extract the DNA used for sequencing. *wspb* and *WD637* are *Wolbachia* markers; *AgRPS7*, *EF* and *18S* are *Aedes spp*. markers (*18S* is a multicopy gene) and *12S* is a mitochondrial marker. The primers used are described in Table S2.

### **Table S2.** Primers used to characterize the bands obtained after density gradient purification of wBol1-b-infected RML12 cells, and to determine the presence and location of secA genes in wBol1-b and wHa.

| **Primer** | **Primer sequence (5’-3’)** | **Target gene** | **Reference** |
| --- | --- | --- | --- |
| wspb 81F  wspb 522R | TGGTCCAATAAGTGATGAAGAAAC  ACCAGCTTTTGCTTGATA | *w*Bol1-b surface protein gene *wsp* | Zhou et al, 1998 |
| 693F  693R | TGTCTGGCGCTAGAAAAG  TTTCGTTTACTTGGCACA | *Wolbachia* ankyrin gene WD0637 | Iturbe-Ormaetxe, pers. comm. |
| AgRPS7-F  AgRPS7-R | GGAGCTGGAGATGAACTCGG  GCAATGAACACGACGTGCTT | Host nuclear gene *RPS7* | Cook, pers. comm. |
| EF-F  EF-R | CCCGCTTCGAGGAAATCAAGAAGGA  CAATGTGAGCGGTGTGGCAATCCA | Host nuclear gene elongase factor | This study |
| 18S-F  18S-R | CTGGTTGATYCTGCCAGT  ACCAGCTTTTGCTTGATA | Host nuclear 18S multicopy gene | Iturbe-Ormaetxe, pers. comm. |
| 12S-AI  12S-BI | AAACTAGGATTAGATACCCTATTAT  AAGAGCGACGGGCGATGTGT | Host mitochondrial 12S gene | Zhou et al, 1998 |
| mutLSecA1-F mutLSecA1-R | GCTTCTCCCCTAAACCCAAG  TTGTCGAAGGAGATGGTGGT | wBol1_1092-1093 boundary | This work |
| SecA2Tran-F SecA2Tran-R | CCTTTCCAGGTATGCTGCTT  CTACTGCCGCCCTGCTATAC | wBol1_1089-1091 boundary | This work |
| 1091-F  1091-R | CTAGATTTTATAGGCAATTCGTGGG  ATTATGTGTTGCTATTCGAAATGACTC | *w*Ha homolog of wBol1_1091 | This work |
| 1092-2  1092-3  1092-4  1092-5  1092-6 | CTAGAGCCTCTATAAATTTCTCC  CTTACCAACAGCTTCTTACTATC  CTCAGCACTGATCACTTTTAGC  GTATTACACCCTTTTAATGGAGCAC  CTTCCACATCACGCTCTTTC | *w*Ha homolog of wBol1_1092 | This work |

**Table S3:** Partial prophage regions of the *w*Bol1-b assembly.

**wBol1_0041 – wBol1_0049** **End of scaffold 3**

wBol1_0041 **Site-specific recombinase, resolvase family**
wBol1_0042 Conserved hypothetical protein
wBol1_0043 **Gp29 protein**
wBol1_0044 Ankyrin repeat domain protein
wBol1_0045 Putative uncharacterized protein **Gp27**
wBol1_0046 **Gp26** protein
wBol1_0047 **Baseplate assembly protein W**, putative
wBol1_0049 Putative phage related protein

**wBol1_0051 – wBol1_0057** **Start of scaffold 4**

wBol1_0051 **Holliday junction resolvasome,** endonuclease subunit
wBol1_0052 Phage related **DNA methylase**wBol1_0053 Putative uncharacterized protein
wBol1_0054 Ankyrin repeat domain protein
wBol1_0055 **Gp29** protein
wBol1_0056 Conserved hypothetical protein
wBol1_0057 **Site-specific recombinase, resolvase family**

**wBol1_0152 – wBol1_0159 End of scaffold 1**

wBol1_0152 repA
wBol1_0153 Hypothetical protein
wBol1_0154 Hypothetical protein
wBol1_0158 **Gp8** protein
wBol1_0159 **Holliday junction resolvasome,** endonuclease subunit

**wBol1_0161 – wBol1_0219 Complete length of scaffold 17**

wBol1_0161 Site-specific recombinase, resolvase family
wBol1_0162 Conserved hypothetical protein
wBol1_0163 Ankyrin repeat domain protein
wBol1_0164 Ankyrin repeat domain protein
wBol1_0165 Putative uncharacterized protein Gp27
wBol1_0166 Baseplate assembly protein GpJ
wBol1_0167 Putative phage protein
wBol1_0168 Similar to probable transmembrane protein
wBol1_0169 Gp24 protein
wBol1_0170 Putative uncharacterized protein Gp8
wBol1_0171 Minor tail protein Z, putative
wBol1_0172 Putative phage related protein
wBol1_0173 Putative phage related protein
wBol1_0174 Hypothetical protein
wBol1_0175 Putative phage portal protein
wBol1_0176 N-acetylmuramoyl-L-alanine amidase, putative
wBol1_0177 Putative phage related protein
wBol1_0178 Phage terminase large subunit GpA
wBol1_0180 Ankyrin domain protein PK1
wBol1_0181 Hypothetical protein WRi_010290
wBol1_0182 Putative membrane protein
wBol1_0183 Phage related DNA methylase
wBol1_0184 Holliday junction resolvasome, endonuclease subunit
wBol1_0186 Hypothetical protein
wBol1_0187 Putative uncharacterized protein
wBol1_0188 Hypothetical protein
wBol1_0189 Hypothetical protein
wBol1_0190 Regulatory protein RepA, putative
wBol1_0192 Hypothetical protein
wBol1_0193 Hypothetical protein WRi_007610
wBol1_0194 Hypothetical protein WD0589
wBol1_0195 Putative uncharacterized protein
wBol1_0196 Hypothetical protein
wBol1_0197 Hypothetical protein
wBol1_0198 Hypothetical protein
wBol1_0199 Putative uncharacterized protein
wBol1_0200 Hypothetical protein Wendoof_01000549
wBol1_0201 Putative phage related protein
wBol1_0202 Hypothetical protein
wBol1_0203 Putative uncharacterized protein
wBol1_0204 Phage major tail sheath protein
wBol1_0205 Phage tail tube protein
wBol1_0206 Putative phage related protein
wBol1_0207 Phage tail tape measure protein
wBol1_0208 Phage tail protein GpU
wBol1_0209 Prophage P2W3, tail protein X, putative
wBol1_0210 Phage late control gene d protein GpD
wBol1_0211 Ankyrin domain protein ank12
wBol1_0212 Putative uncharacterized protein
wBol1_0213 Patatin family protein
wBol1_0214 Transcriptional regulator, putative
wBol1_0215 Transcriptional regulator, putative
wBol1_0216 Hypothetical protein WD0256
wBol1_0218 Hypothetical protein Wendoof_01000194
wBol1_0219 Putative dna repair protein radc

**wBol1_0220 – wBol1_0235 Complete length of scaffold 7**

wBol1_0220 Putative phage related protein
wBol1_0221 Putative phage related protein
wBol1_0222 Hypothetical protein
wBol1_0223 Hypothetical protein Wendoof_01000382
wBol1_0224 Phage major tail sheath protein
wBol1_0225 Phage tail tube protein
wBol1_0226 Putative uncharacterized protein
wBol1_0227 Putative phage related protein
wBol1_0228 Phage-related tail protein
wBol1_0229 Phage tail protein GpU
wBol1_0230 Phage tail protein GpX
wBol1_0231 Phage late control gene d protein GpD
wBol1_0232 Ankyrin repeat domain protein
wBol1_0233 Putative uncharacterized protein
wBol1_0234 Hypothetical protein Wendoof_01000458

**wBol1_0237 to wBol1_0248 Start of scaffold 2**

wBol1_0237 Hypothetical protein Wendoof_01000458
wBol1_0238 Hypothetical protein
wBol1_0239 Gp3 protein
wBol1_0240 Transposase
wBol1_0241 Hypothetical protein
wBol1_0242 Hypothetical protein
wBol1_0243 Hypothetical protein
wBol1_0244 Transposase, IS5 family, truncation
wBol1_0245 Hypothetical protein
wBol1_0246 Gp32 protein
wBol1_0247 Hypothetical protein
wBol1_0248 Site-specific recombinase, resolvase family

**wBol1_1097 – wBol1_1111** **One end of scaffold 20**

wBol1_1097 Phage related **DNA methylase**
wBol1_1098 Hypothetical protein WRi_010290
wBol1_1099 Ankyrin repeat domain protein
wBol1_1100 Hypothetical protein
wBol1_1101 Phage uncharacterized protein
wBol1_1103 Putative phage **portal protein**
wBol1_1104 **Orf7** protein
wBol1_1105 Conserved hypothetical protein
wBol1_1106 Putative phage related protein
wBol1_1108 Putative minor **tail protein Z**
wBol1_1109 Putative uncharacterized protein Gp8
wBol1_1110 Putative **baseplate assembly protein GpV**
wBol1_1111 **Gp25** protein

**wBol1_1345 – wBol1_1371** **Other end of scaffold 20**

wBol1_1345 **Recombinase** family
wBol1_1348 **Gp29** protein
wBol1_1349 Ankyrin repeat domain protein
wBol1_1350 Putative uncharacterized protein Gp27
wBol1_1352 **Gp26** protein
wBol1_1353 Putative uncharacterized protein **GpW**
wBol1_1354 Putative phage related protein
wBol1_1355 Putative **baseplate assembly protein GpV**
wBol1_1356 Putative uncharacterized protein
wBol1_1357 **Minor tail protein Z**, putative
wBol1_1358 Putative uncharacterized protein
wBol1_1359 Putative phage related protein
wBol1_1360 Putative uncharacterized protein
wBol1_1361 Putative **minor capsid protein c**
wBol1_1362 Putative **phage portal protein**
wBol1_1364 Hypothetical protein WRi_010260
wBol1_1365 **Phage terminase large subunit GpA**
wBol1_1367 Ankyrin domain protein PK1
wBol1_1368 Putative phage related protein
wBol1_1369 Putative membrane protein
wBol1_1370 Phage related **DNA methylase**wBol1_1371 **Holliday junction resolvasome**, endonuclease subunit

**wBol1_1372 – wBol1_1378 Complete length of scaffold 13**

wBol1_1372 Regulatory protein RepA, putative
wBol1_1373 Hypothetical protein WD0583
wBol1_1375 Conserved hypothetical protein
wBol1_1376 Hypothetical protein WD0589
wBol1_1377 Hypothetical protein Wendoof_0100092
wBol1_1378 Hypothetical protein WD0591

**Table S4, list of putative *w*Bol1-b-specific genes.**

Genes were considered to be *w*Bol1-b-specific if (a) they were not clustered in the orthoMCL analysis, and (b) when used as a blastp query vs the NR database with E-value cut-off of 10, they had either no hit, or the best hit was a non-*Wolbachia* gene. Annotations are given below for NR hits if the E-value was better than 1e-5; each of these nine genes is discussed in the main text.

| **wBol1-b gene name** | **Gene length in aa** | **Annotation of best NR blast hit, if present** |
| --- | --- | --- |
| wBol1_0035 | 168 | conserved hypothetical protein [Legionella longbeachae D-4968] |
| wBol1_0058 | 52 | no NR hit |
| wBol1_0072 | 49 | no NR hit |
| wBol1_0074 | 44 | no NR hit |
| wBol1_0093 | 326 | transposase [Rhodobacteraceae bacterium KLH11] |
| wBol1_0153 | 52 | no NR hit |
| wBol1_0186 | 62 | no NR hit |
| wBol1_0187 | 340 | hypothetical protein Mbur_1214 [Methanococcoides burtonii DSM 6242] |
| wBol1_0189 | 41 | no NR hit |
| wBol1_0255 | 42 | no NR hit |
| wBol1_0256 | 441 | hypothetical protein SINV_00084 [Solenopsis invicta] |
| wBol1_0257 | 116 | hypothetical protein SINV_00084 [Solenopsis invicta] |
| wBol1_0259 | 83 | no NR hit |
| wBol1_0260 | 42 | no NR hit |
| wBol1_0261 | 112 | no NR hit |
| wBol1_0262 | 184 | radical SAM domain-containing protein [Micromonospora aurantiaca ATCC 27029] |
| wBol1_0265 | 414 | radical SAM domain-containing protein [Micromonospora aurantiaca ATCC 27029] |
| wBol1_0270 | 62 | no NR hit |
| wBol1_0283 | 52 | no NR hit |
| wBol1_0317 | 47 | no NR hit |
| wBol1_0373 | 49 | no NR hit |
| wBol1_0448 | 40 | no NR hit |
| wBol1_0503 | 51 | no NR hit |
| wBol1_0506 | 51 | no NR hit |
| wBol1_0514 | 52 | no NR hit |
| wBol1_0570 | 301 | no NR hit |
| wBol1_0647 | 51 | no NR hit |
| wBol1_0693 | 94 | no NR hit |
| wBol1_0754 | 49 | no NR hit |
| wBol1_0766 | 54 | no NR hit |
| wBol1_0788 | 50 | no NR hit |
| wBol1_0811 | 42 | no NR hit |
| wBol1_0820 | 40 | no NR hit |
| wBol1_0856 | 48 | no NR hit |
| wBol1_0924 | 46 | no NR hit |
| wBol1_1026 | 88 | no NR hit |
| wBol1_1090 | 49 | no NR hit |
| wBol1_1091 | 1495 | hypothetical protein AaeL_AAEL001543 [Aedes aegypti] |
| wBol1_1092 | 3942 | Protein translocase subunit secA [Harpegnathos saltator] |
| wBol1_1174 | 40 | no NR hit |
| wBol1_1186 | 81 | no NR hit |
| wBol1_1220 | 76 | no NR hit |
| wBol1_1319 | 45 | no NR hit |
| wBol1_1331 | 40 | no NR hit |

**Figure S2** Maximum likelihood phylogenetic trees of three *w*Bol1-b genes putatively horizontally transferred from other bacterial groups. (a) wBol1_0093, (b) wBol1_0035, (c) wBol1_0187. Bootstrap values over 50 are shown. *Wolbachia* genes are indicated with an arrowhead.

(a)


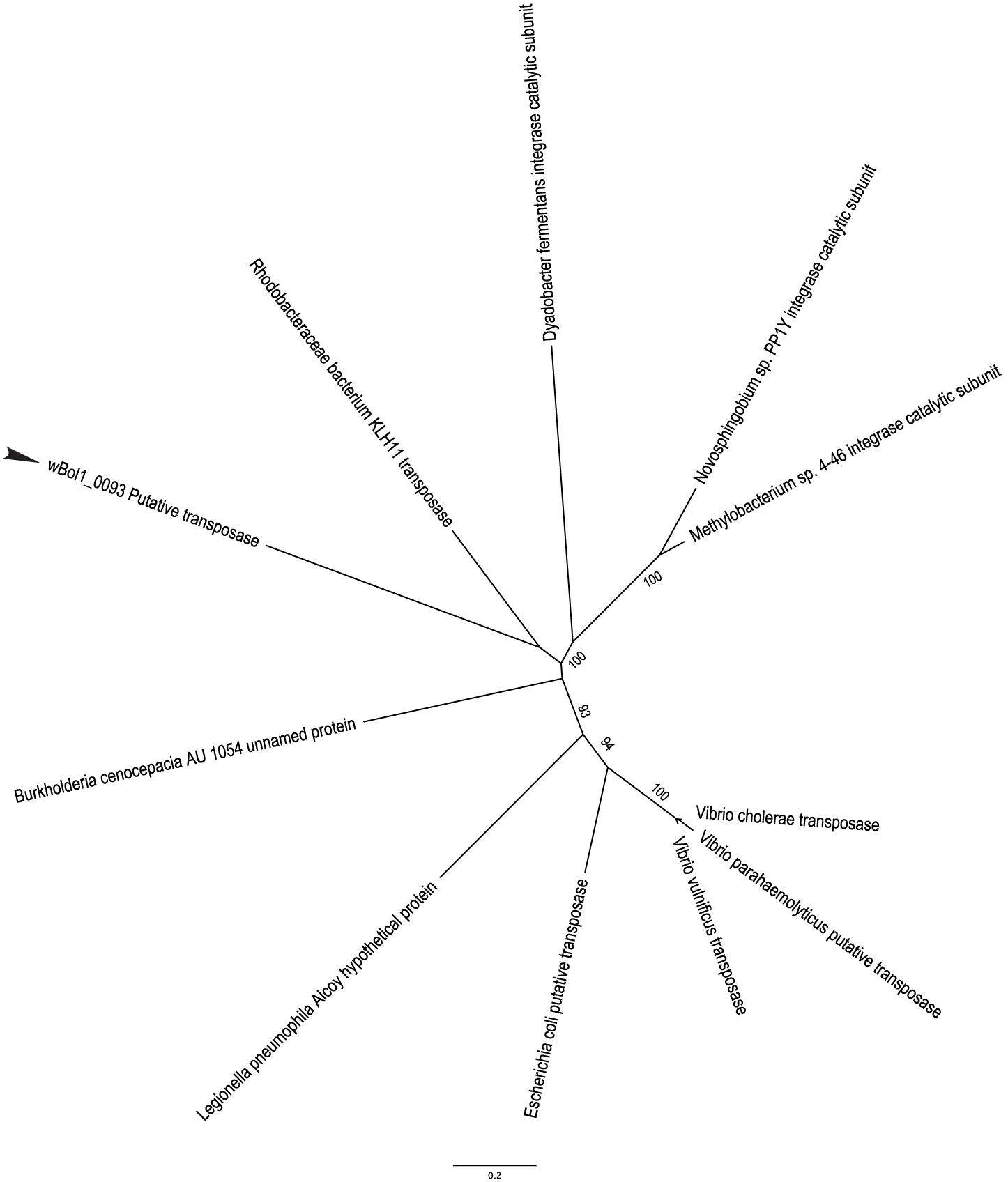
(b)


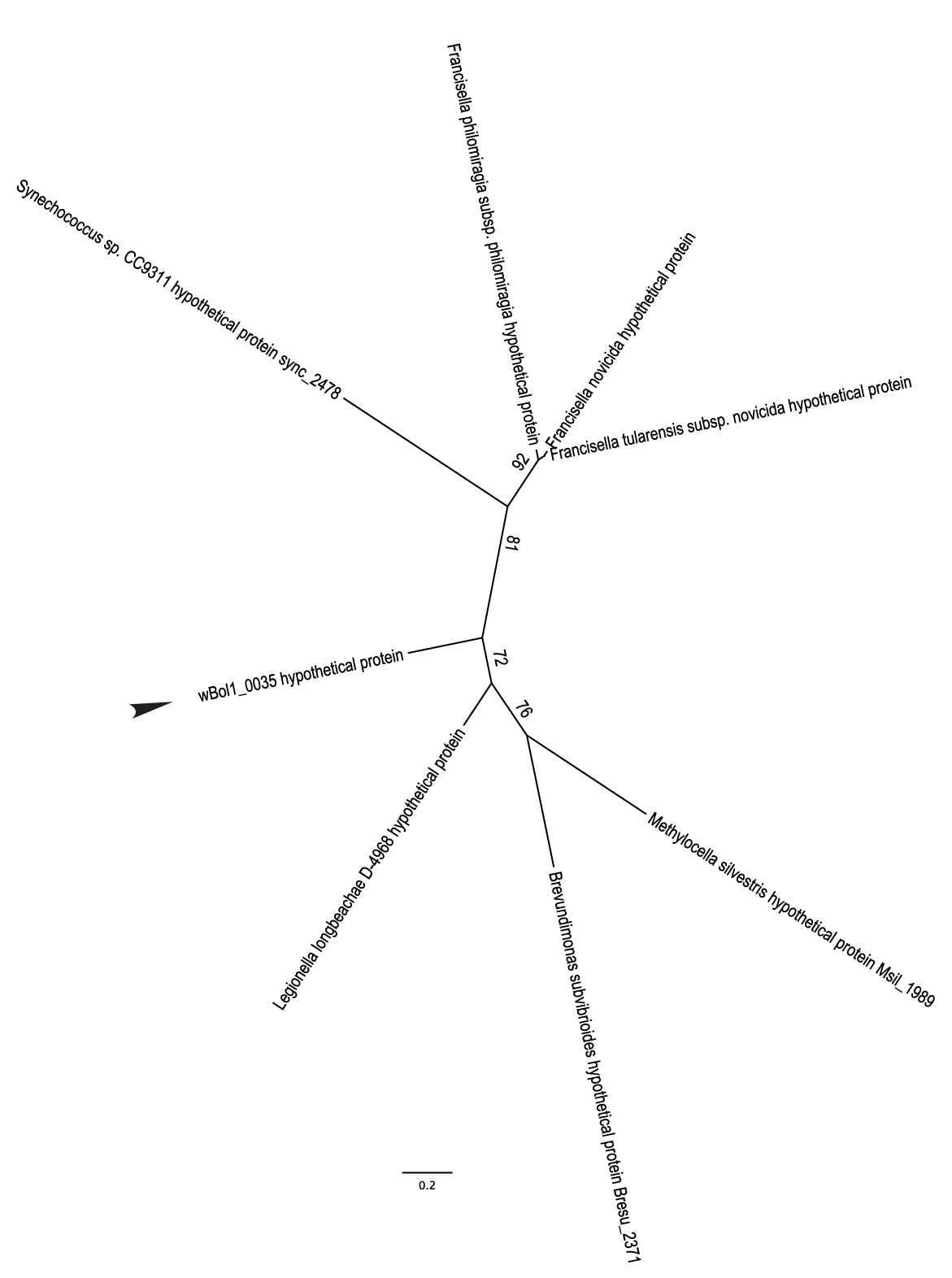


(c)


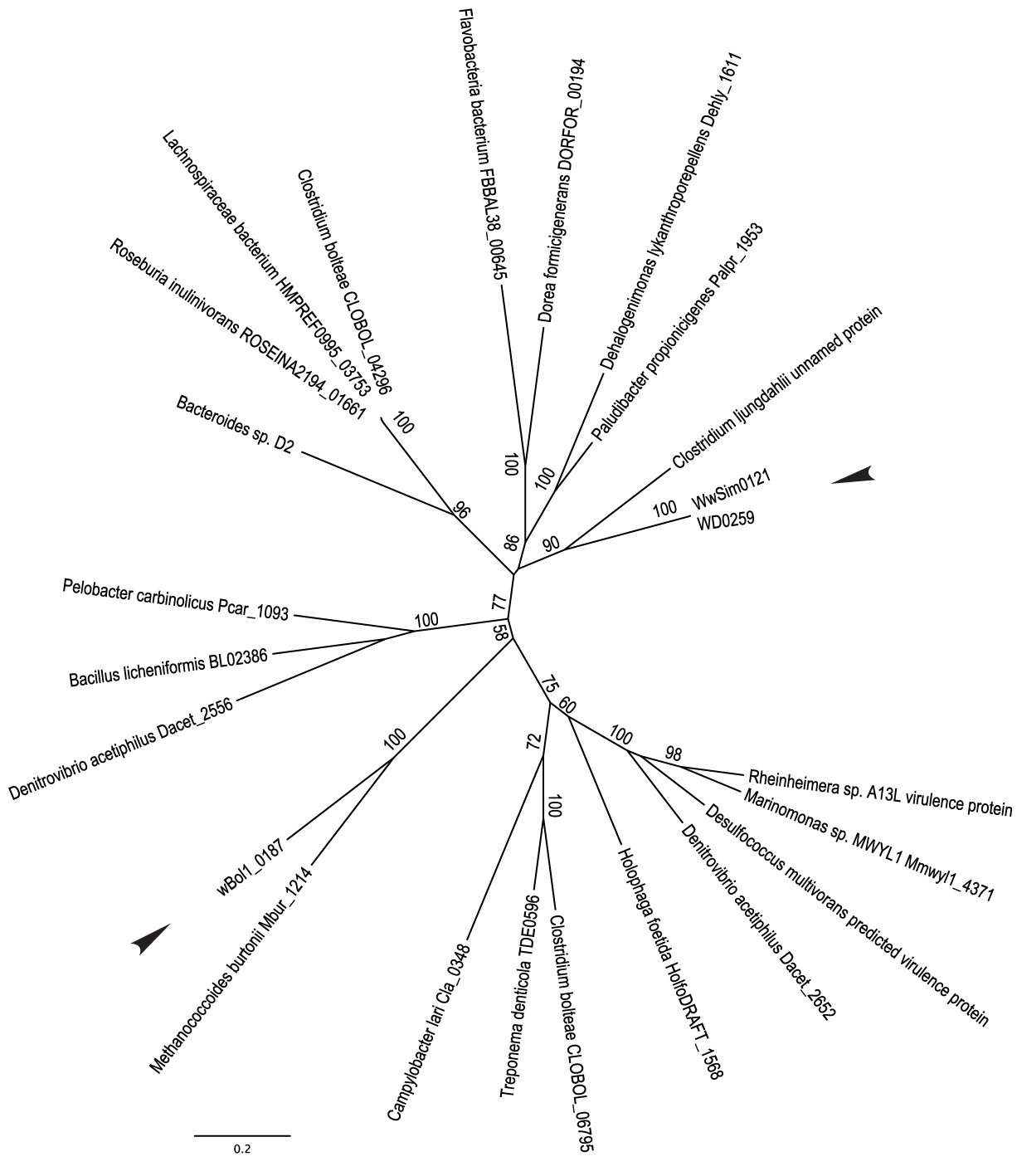
**Figure S3** Maximum likelihood phylogenetic tree of wBol1_1092 including partial sequences of the *w*Ha ortholog.


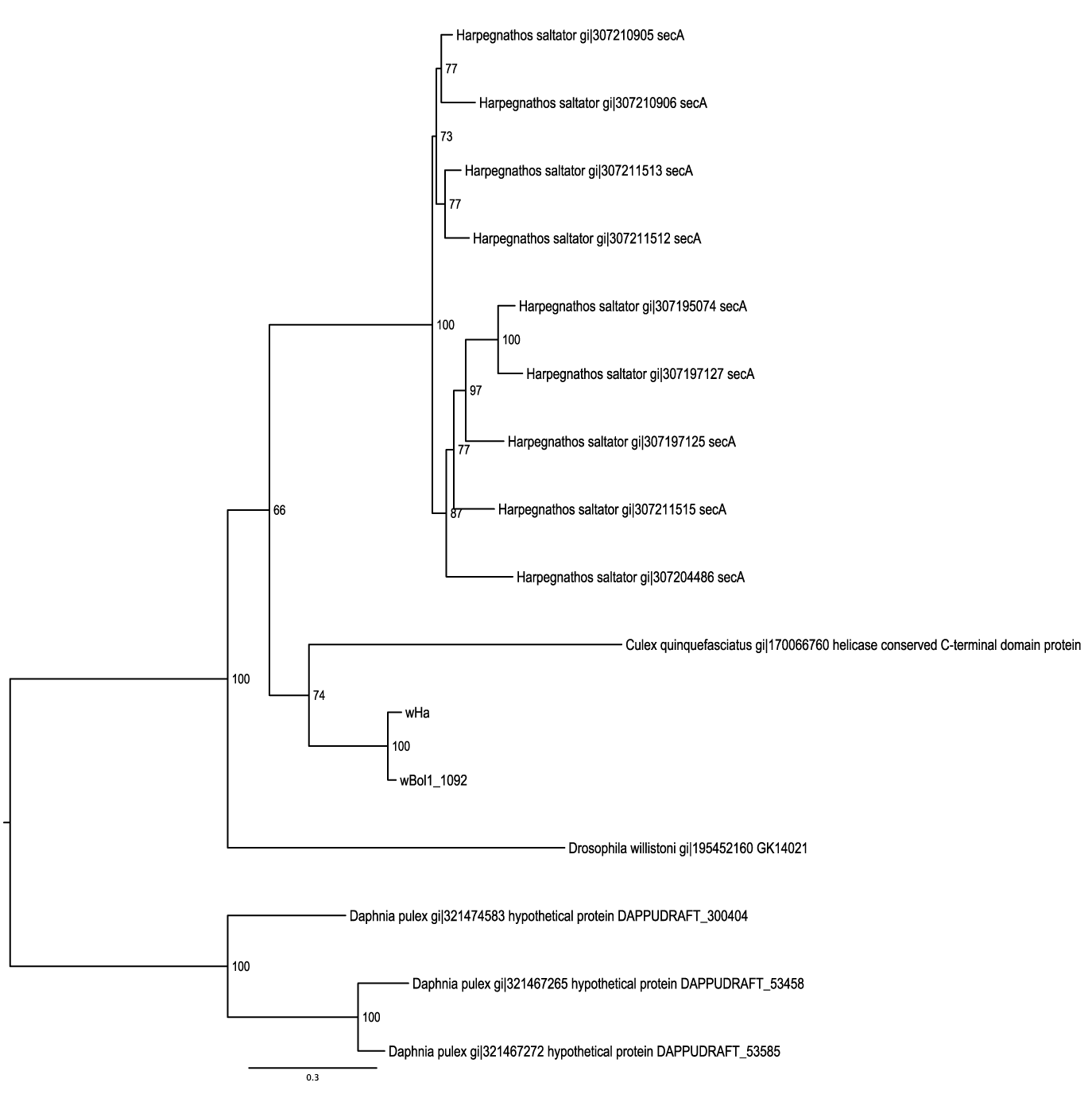


**Introns in eukaryotic secA genes**

The *C. quinquefasciatus* gene most closely related to wBol1_1092, CPIJ018005, is annotated with introns, but on closer inspection it appears to consist of two adjacent paralogs, and the two introns in each copy either translate in frame or translate with one to two frameshifts, and are therefore likely to be the result of degeneration of a single exon rather than true introns. The closely related *D. willistoni* gene, Dwil_GK14021, and most of the *H. saltator* secA genes are annotated without introns; those that do have annotated introns have intron sequences that match exonic sequence of other secA gene copies in the genome, and translate with a small number of frameshifts – these also appear to be degenerating copies of single-exon genes rather than genes with genuine introns. A similar pattern is seen in *A. aegypti* and *C. quinquefasciatus* genes homologous to wBol1_1091: they are either annotated without introns, or their introns appear to be degenerate exonic sequence.

**Transcriptional direction of WD1302**

While checking agreement between different ortholog prediction methods, we noticed that one core gene ortholog group was predicted by a method that relied on nucleotide sequence, but not by protein-based methods. Based on nucleotide similarity and syntenic conservation, genes WD1302, WRi_013310, WPa_1060, wBol1_0867 and Wbm0388 are clearly orthologous. However, the gene has been annotated as transcribed from different strands in different genomes: one way in *w*Mel, and the other in *w*Ri, *w*Pip and *w*Bm.

No expression data are available for any of these genes, to our knowledge. However, the protein predicted in the *w*Ri, *w*Pip and *w*Bm genomes contains a putative conserved multiple resistance and pH regulation protein F superfamily domain, while the protein predicted in the *w*Mel genome contains no recognized conserved domains. Moreover, using the proteins predicted from each of the strands as blastP queries against the NR database shows that WD1302 has no significant hits other than a hypothetical protein in the JHB *w*Pip genome, while WRi_013310, WPa_1060, wBol1_0867 and Wbm0388 each have significant hits to multiple annotated proteins in other bacterial taxa. We think it is likely that this represents an incorrect gene prediction in the *w*Mel genome.

References

1. Frentiu FD, Robinson J, Young PR, McGraw EA, O'Neill SL: **Wolbachia-Mediated Resistance to Dengue Virus Infection and Death at the Cellular Level**. *PLoS One* 2010, **5**(10).

2. Heddi A, Grenier AM, Khatchadourian C, Charles H, Nardon P: **Four intracellular genomes direct weevil biology: Nuclear, mitochondrial, principal endosymbiont, and Wolbachia**. *PNAS* 1999, **96**(12):6814-6819.
